# Supplementary material for: Predicting congenital syphilis cases: A performance evaluation of different machine learning models
Source: PLoS One. 2023 Jun 2;18(6):e0276150. doi: 10.1371/journal.pone.0276150 (PMC10237406; doi:10.1371/journal.pone.0276150)
Supplement: Attachment — Submitted filename: Plos one syphilis reviewer.pdf [file pone.0276150.s001.pdf]

PONE-D-22-27034
PLOS ONE
Predicting congenital syphilis cases: a performance evaluation of different machine learning models

Reviewer #1: The paper was written fairly well, however several issues need to be addressed to consider as a good technical paper.
Dear reviewer, we do appreciate your comments and suggestions to improve our work. We have addressed them properly.

Abstract
The number of dataset is not defined here as well as the technique of feature selection.
In Section Data set, we described the number of data set and tables as follow:
"We used nine tables with anonymized data provided by the PMCP, extracted from their information system, named SIS-MC. These tables contain clinical and sociodemographic data regarding antenatal care, pregnant women's outcomes, and their children, from the cities served by the PMCP in the State of Pernambuco, Brazil, between the years of 2013 and 2021 [...]".
"Fig 1 illustrates the data pre-processing methodology designed to unify the nine tables provided by the PMCP into an unified dataset [...]".

In the Section Data set, we updated Fig 1 to illustrate the unification of the multiple tables into a unique data set, called a unified data set. We also updated the URL of the pre-processed data set publicly available at Data Mendeley.

In Section SFA models, we said:
"In order to automatically find the best subset of attributes (feature selection) and the best model configuration (hyperparameter optimization), in a given search space, two techniques were applied: (i) SFA and(ii) grid search, respectively. For each combination of the grid search technique (Table 3 presents the hyperparameters used in the grid search), two flavors of the SFA technique were performed (SFS and SBS)."

In Section Hyper-parameter optimization and feature selection techniques, we have described both techniques:
"The SFS flavor starts with an empty subset of attributes; with each iteration, a new attribute is added, thereby selecting the attributes that increase the model's performance. On the other hand, the SBS flavor begins with a subset including all the attributes of the data set; with each iteration, different combinations of attributes are evaluated, and the attribute with the least impact on model performance is removed."

The results defined was in general, without including statistical results.
As we did not make repetitions in our experiments, we can not include statistical results in this current work. In total, we had 126 models that were trained using AWS resources and took too long to be finished (more than 5 months). Therefore, due to the time needed to retrain the models and understanding the relevance of this question, we have added statistical analysis as future work.

Result and Discussion

The result obtain from the study is too low compared to reference [7].

We did not understand this point. Our reference [7] is an nationwide epidemiological bulletin from the Brazilian Ministry of Health. We are not able to compare results, as our dataset is restricted to specific samples in only one state (Pernambuco) and [7] does not have prediction results.
