# Supplementary material for: Predicting congenital syphilis cases: A performance evaluation of different machine learning models
Source: PLoS One. 2023 Jun 2;18(6):e0276150. doi: 10.1371/journal.pone.0276150 (PMC10237406; doi:10.1371/journal.pone.0276150)
Supplement: Attachment — Submitted filename: Plos one syphilis reviewer 2.pdf [file pone.0276150.s002.pdf]

PONE-D-22-27034
PLOS ONE
Predicting congenital syphilis cases: a performance evaluation of different machine learning models

Reviewer #1: The author state that table 3 in refer to "the hyperparameters used in the grid search", however in the manuscript table 3 caption is "Baseline characteristics of the data set.". Please make an amendment for this table caption

Dear reviewer, we do appreciate your comment and suggestions to improve our work.

The overall baseline characteristics of the pre-processed data set related to the pregnant women assisted by the PMCP are presented in Table 3; and Table 4 presents the hyperparameters used in the grid search.

We changed the caption of Table 4 and the text that refers to it, changing the word "parameter" to "hyperparameter":

"Hyperparameters used in the grid search."
"Table 4 presents the hyperparameters used in the grid search"
